# Prenatal diagnosis of fetal intraabdominal extralobar pulmonary sequestration: a 12-year 3-center experience in China

**DOI:** 10.1038/s41598-018-37268-1

**Published:** 2019-01-30

**Authors:** Ganqiong Xu, Jiawei Zhou, Shi Zeng, Ming Zhang, Zhu Ouyang, Yili Zhao, Hongxia Yuan, Lili Tong, Chan Yin, Qichang Zhou

**Affiliations:** 1 Department of Ultrasound Diagnosis, The Second Xiangya Hospital, Central South University, Changsha, Hunan 410011 China; 2 Department of Ultrasound, Changsha Hospital for Maternal & Child Health Care, Changsha, China; 3 Department of Ultrasound, The Maternal and Child Health Hospital of Changde City, Changde, China

## Abstract

To provide useful information for diagnosing and predicting fetal intraabdominal extralobar pulmonary sequestration (IEPS), a retrospective review of diagnostic approaches was conducted. Ultrasonography was performed serially in 21 fetuses with IEPS from 2005 to 2017. Prenatal sonographic features, treatment, and outcomes of each case were evaluated and collected. These cases of IEPS were also compared to 43 cases previously reported by other researchers from 1986 to 2017. Of the 21 sonographic features, 14 (67%) were hyperechoic, 21 (100%) were well circumscribed, and 17 (81%) depicted a mass that shifted with fetal breaths/hiccups non-synchronized with adjacent organs (sliding sign). Feeding arteries were detected prenatally in 18 patients (86%). The lesion volume was 10.17 ± 4.66 cm^3^, the congenital cystic adenomatoid malformation volume ratio and cardiothoracic ratio were in normal range. The gestational age at diagnosis, location and echotexture of the lesion, and rate of surgical treatment were similar to previous studies, but with a significantly higher rate of detected feeding arteries (*P* < 0.01), and associated anomalies (*P* < 0.01). All infants who underwent surgery after birth had satisfactory outcomes. The sliding sign and feeding artery are essential features of IEPS in prenatal diagnosis.

## Introduction

Intraabdominal extralobar pulmonary sequestration (IEPS) is a rare type of pulmonary sequestration (PS). PS is a spectrum of bronchopulmonary foregut anomalies, characterized overall as non-functional pulmonary tissue that is sequestered or separated from normal bronchial connections. The prevalence is between 0.15 and 1.8%^1–3^. There are 2 types of PS that have been previously determined: intralobar sequestrations (ILS; 75%), and extralobar sequestrations (ELS; 25%). In ILS, the sequestration shares the common pleura with the normal lung; in ELS, the sequestration has its own pleural envelope^4,5^. Less than 10% of ELS, including IEPS, are reportedly out of the thorax^6–8^.

The first prenatal diagnosis of IEPS was reported in 1986^9^, and to the best of our knowledge, only 43 cases of IEPS had been reported in the literature. Appropriate prenatal diagnosis of IEPS is important to optimize the management strategy for affected neonates. However, most previous publications either had a small study population (no more than 6 cases) or were merely single case reports. Furthermore, most previous studies did not describe the details of sonographic features of IEPS, congenital cystic adenomatoid malformation volume ratio, or cardiothoracic ratio. Due to the lack of studies with a larger sample size, the prenatal sonographic features, natural course, and perinatal outcomes have not been discussed definitively.

This study describes diagnostic approaches toward fetal IEPS, especially ultrasound examinations, and clinical outcomes.

## Results

### General conditions of the present study

Our 3 prenatal sonographic diagnostic centers performed 210,237 prenatal ultrasound exams. Of these, there were 21 cases of IEPS, confirmed by autopsy or surgery pathology (Table 1).

**Table 1:** Clinical & ultrasound imaging features of patient with IEPS.

	Birth y	Prenatal ultrasound	Associated anomalies	Postnatal imaging	Treatment	Follow-up
1	2005	24 wk, L suprarenal mass; 30 wk, increased size from 2.7 × 2.3 to 4.2 × 3.3 cm	No	No	Abortion	—
2	2006	21 wk, L suprarenal mass, 3.0 × 2.8 cm	DH	No	Abortion	—
3	2007	21 wk, R suprarenal mass, 2.7 × 1.9 cm	No	US through 5 mo: 3.5 × 2.3 cm	Open surgery at 55 mo (2012)	Serial US through 55–67 mo (continue 12 mo), NED
4	2008	26 wk, L suprarenal mass, 3.0 × 2.0 cm	DH	No	Abortion	—
5	2009	25 wk, L suprarenal mass, 2.6 × 2.2 cm	No	US through 2 mo, CT through 20 mo: 3.5 × 3.0 cm	Open surgery at 45 mo (2013)	Serial US through 47–60 mo (continue 13 mo), NED
6	2009	26 wk, L suprarenal mass, 3.5 × 3.0 cm	DH	No	Abortion	—
7	2009	26 wk, L suprarenal mass, 4.0 × 3.0 cm	DA	No	Abortion	—
8	2010	24 wk, L suprarenal mass 2.7 × 2.6 cm	DH	No	Abortion	—
9	2010	23 wk, R suprarenal mass 4.6 × 2.9 cm	No	US through 4 mo, CT through 24 mo: 4.7 × 3.0 cm	Open surgery at 38 mo (2014)	Serial US through 38–50 mo (continue 12 mo), NED
10	2011	21 wk, L suprarenal mass; 28 wk, increased size from 2.0 × 1.9 cm to 3.5 × 2.9 cm	No	No	Abortion	—
11	2011	27 wk, L suprarenal mass; trisomy 18, 3.0 × 3.0 cm	AC & DH	No	Abortion	—
12	2012	22 wk, L suprarenal mass 4.1 × 3.0 cm	No	US through 2 mo, CT through 16 mo: 4.7 × 3.5 cm	Open surgery at 25 mo (2014)	Serial US through 25–35 mo (continue 10 mo), NED
13	2012	25 wk, R suprarenal mass 3.0 × 1.9 cm	No	US through 4 mo, CT through 6 mo: 4.2 × 2.9 cm	Open surgery at 24 mo (2014)	Serial US through 24–36 mo (continue 12 mo), NED
14	2013	27 wk, L suprarenal mass 2.7 × 2.3 cm	No	US through 4 mo, MRI through 12 mo: 3.0 × 2.5 cm	LAP at 20 mo (2015)	Serial US through 20–31 mo (continue 11 mo), NED
15	2013	22 wk, L suprarenal mass 3.2 × 3.1 cm	DH	No	Abortion	—
16	2014	24 wk, L suprarenal mass 3.5 × 2.7 cm	No	US through 1 mo, MRI through 10 mo: 4.7 × 3.3 cm	LAP at 16 mo (2015)	Serial US through 16–26 mo (continue 10 mo), NED
17	2015	26 wk, L suprarenal mass, 3.9 × 2.9 cm	No	US through 3 mo: 4.5 × 3.3 cm	LAP at 13 mo (2016)	Serial US through 14–25 mo (continue 11 mo), NED
18	2015	25 wk, L suprarenal mass, 3.0 × 2.3 cm	No	US through 2 mo, 3.5 × 2.7 cm	LAP at 8 mo (2016)	Serial US through 8–19 mo (continue 11 mo), NED
19	2016	24 wk, L suprarenal mass, 3.5 × 3.2 cm	No	US through 5 d, CT through 4 mo: 4.5 × 3.9 cm	LAP at 6 mo (2016)	Serial US through 6–15 mo (continue 9 mo), NED
20	2016	29 wk, L suprarenal mass, 2.9 × 2.6 cm	No	US through 10 d, 3.0 × 2.8 cm	LAP at 3 mo (2016)	Serial US through 3–13 mo (continue 10 mo), NED
21	2017	21 wk, L suprarenal mass, 3.0 × 2.4 cm		US through 7 d, MRI through 1 mo: 3.5 × 2.9 cm	LAP at 2 mo (2017)	Serial US through 2–3 mo (continue 1 mo), NED

DH, diaphragmatic hernia; DA, duodenal atresia; AC, aortic coarctation; LAP, laparoscopy; NED, no evidence of disease.

### Postmortem findings or surgery findings in the present study

**Figure 1:**
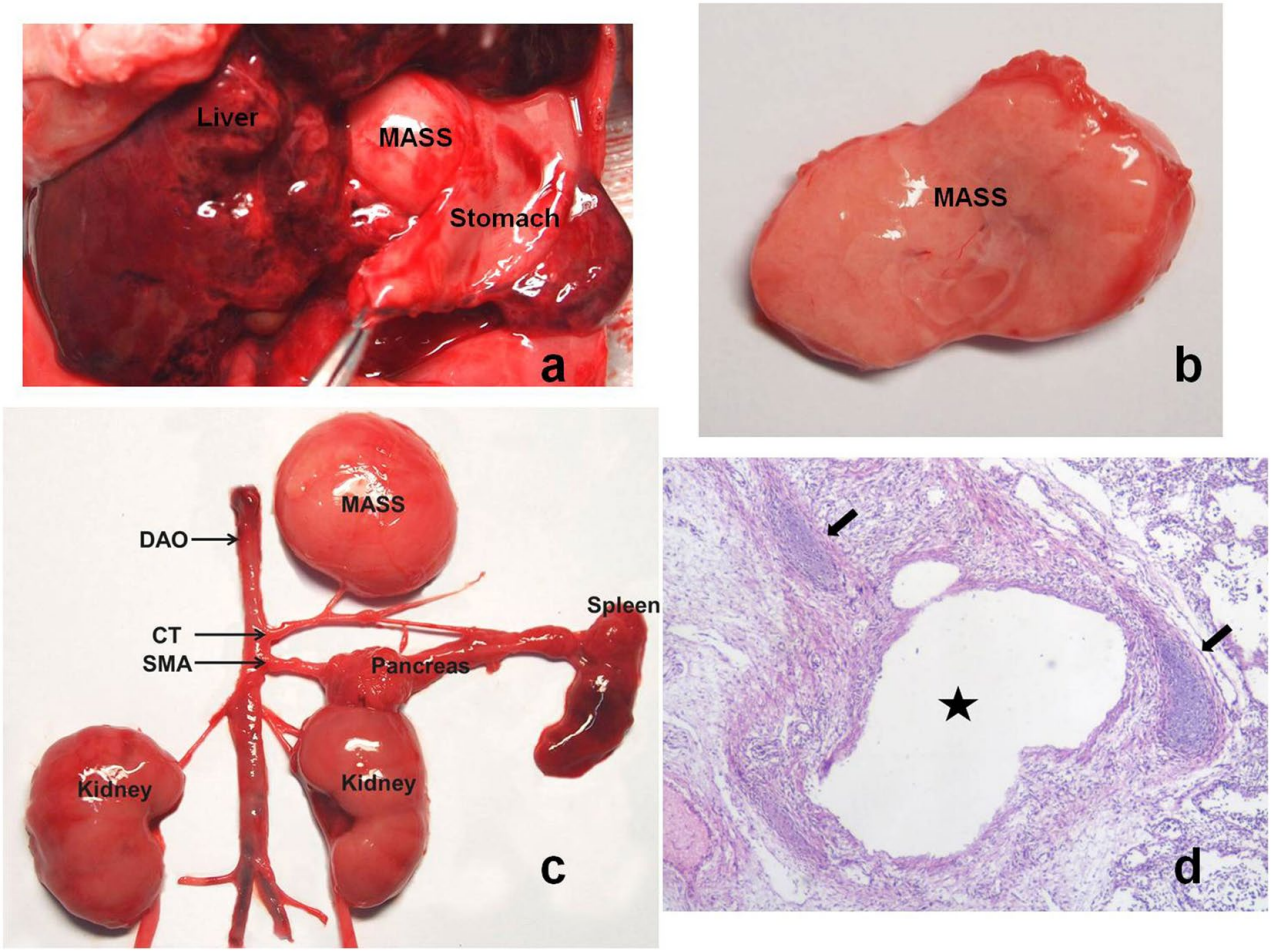
IEPS in autopsy and histopathology. (**a**) Autopsy showing a soft, solid mass at upper abdomen with no adherence to its surrounding structures and no communication to the gastrointestinal tract. (**b**) The cut surface of the mass showing spongy structure. (**c**) Autopsy showing a mass with a feeding vessel arising from the celiac trunk (CT), which is the first visceral branch of the descending aorta (DAO). SMA, superior mesenteric artery. (**d**) The mass containing mixed dysplastic and hyperplastic lung tissue, with bronchiole-like structures (asterisk) and cartilage (arrows) on histopathology. (H&E stain, ×40).

Abortion was performed in 43% (9/21) of the patients with IEPS, according to the preferences of the parents who did not want to suffer potential adverse outcomes. Seven of the 9 aborted fetuses had concomitant anomalies and 2 had a mass size that had grown larger since the ultrasound examination. Postmortem autopsy confirmed all findings on prenatal ultrasound. There were soft, solid masses with no adherence to the surrounding structures and no communication to the gastrointestinal tract (Fig. 1a,b). The feeding arteries were from the abdominal aorta or its branch such as the celiac trunk (Fig. 1c). Final histological evaluation confirmed a diagnosis of IEPS in all cases (Fig. 1d).

**Figure 2:**
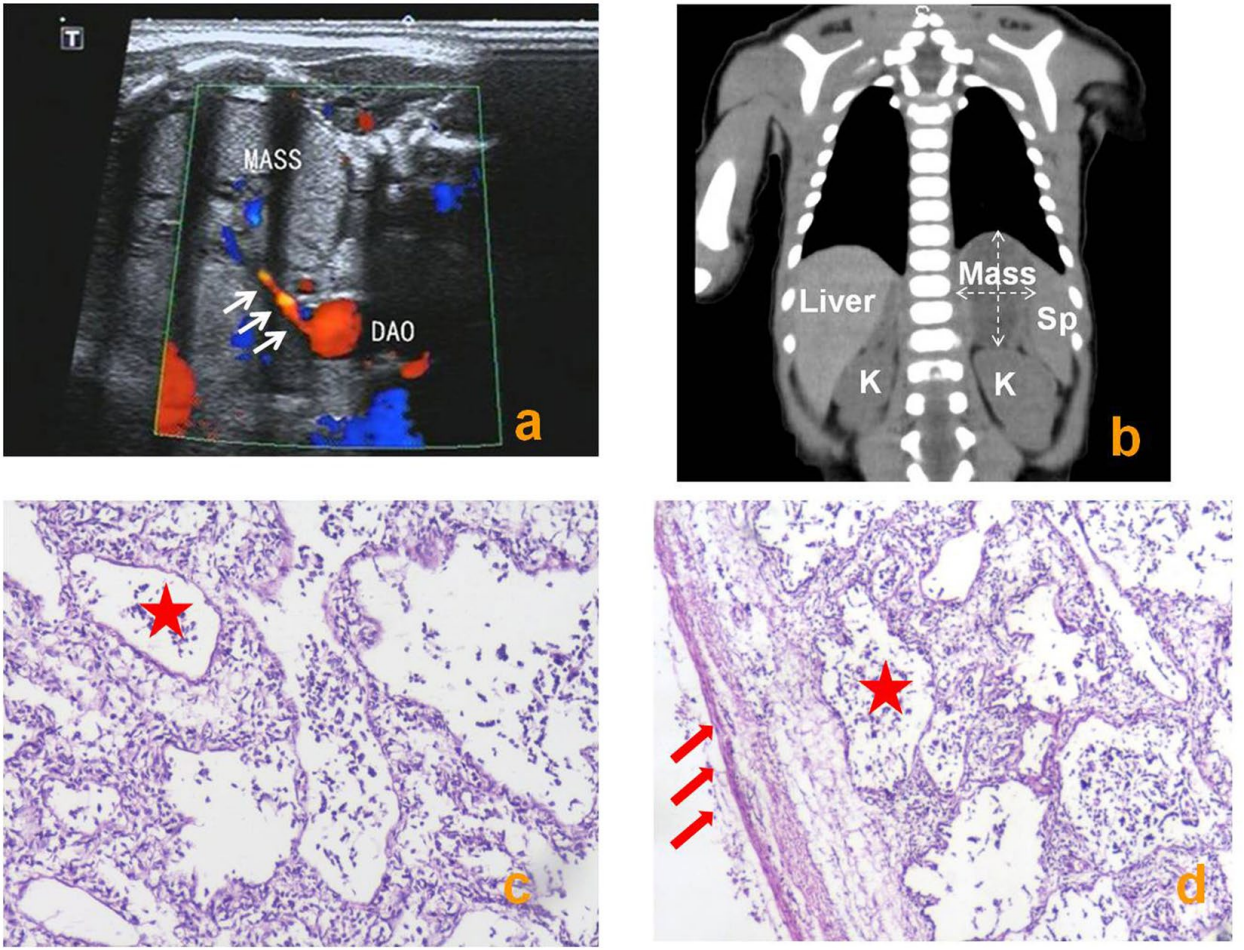
IEPS in postnatal imagings and histopathology. (**a**) Postnatal color Doppler ultrasound showing a mass at the left adrenal area with the feeding artery (arrows) arising from the descending aorta (DAO). (**b**) CT scans showing an ellipsoidal low-density mass at the left adrenal area. This mass was delineated by a clear boundary, and CT value was approximately 35 HU. The left side of the diaphragm was pushed upwards. Sp, spleen; K, kidney. (**c**,**d**) Postoperative histopathology showing alveolar tissue (asterisk), surface covered with mesothelial cells (arrows) (H&E stain, ×40).

The 12 fetuses that were not aborted (isolated IEPS) survived through birth. After birth, the neonates received imaging exams. Postnatal ultrasound exam was performed in all of these patients. Some lesions showed no change in size and others grew proportionally. The sonographic features of the prenatal and postnatal ultrasound (Fig. 2a) exams were similar. Postnatal computed tomography (CT; Fig. 2b) or magnetic resonance imaging (MRI) was performed in some patients, which were consistent with the prenatal ultrasound findings.

The mass was successfully removed by surgery in all 12 infants, with 5 and 7 by open and laparoscopic surgery, respectively. Ten of these lesions could be clearly separated from the adjacent organs and others had mild fibrous connection to the adrenal gland. These tumors could be easily excised after finding the tissue stalk containing the supplying vessels arising from the abdominal aorta. Postoperative pathological diagnosis revealed bronchial-alveolar tissue, with the surface covered with mesothelial cells (Fig. 2c,d), consistent with all prenatal diagnoses. All of the patients recovered uneventfully and with favorable outcomes (that is, good recovery, and lack of complications or infection or respiratory or digestive symptoms during the follow-up period). The laparoscopic approach resulted in less postoperative pain and smaller scars. The mean age at surgery was 21.3 ± 17.1 months (range 2–55 mo) and the mean duration of postoperative follow-up was 10.2 ± 3.1 months (range 1–13 mo).

### Essential prenatal ultrasound feature of IEPS in the present study

**Figure 3:**
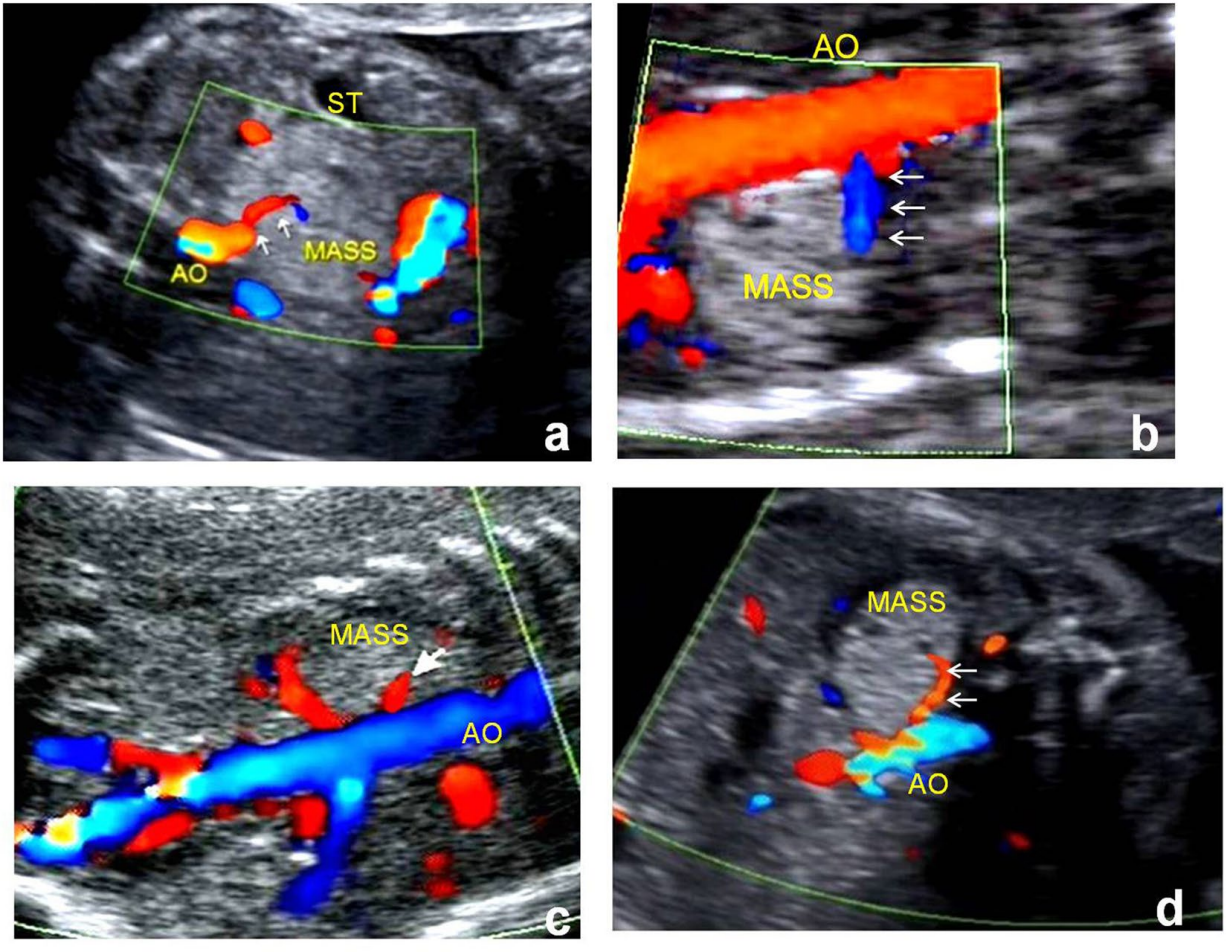
Feeding vessel of IEPS in color Doppler of prenatal US. Color Doppler ultrasound showing masses with the feeding artery (arrows) arising from the abdominal aorta (AO). ST, stomach.

IEPS was more common in the left suprarenal region and appeared as a homogenous and hyperechoic solid mass with ellipsoidal shape (Table 2). Mostly lesions were well circumscribed and displayed a sliding sign (defined as a mass that shifts during fetal breath movements or hiccups, and movements are not synchronized with that of adjacent organs) and feeding artery (Fig. 3). The heart size, cardiothoracic ratio (CTR), and congenital cystic adenomatoid malformation volume ratio (CVR) were in normal range. One-third (33%) of the patients also had other anomalies and none developed hydrops.

**Table 2:** Prenatal IEPS ultrasound imaging features in 21 patients.

Volume, cm^3^	10.17 ± 4.66
Left side	18 (86%)
Hyperechoic	14 (67%)
Ellipsoidal shape	16 (76%)
Well-circumscribed	21 (100%)
Sliding sign	17 (81%)
Associated anomalies	7 (33%)
Hydrops	0 (0)
Detection of feeding artery	18 (86%)
Heart area Z-score	−0.02 ± 0.33
CVR	0.44 ± 0.19
CTR	0.29 ± 0.05

### Variability analysis

The intra-observer and inter-observer variability is reflected by the mean percentage error (Table 3). In the 3 centers, the intra-observer mean percentage error was less than 8.0% for volume, 9.0% for heart area, 8.0% for CVR, and 9.0% for CTR. Inter-observer mean percentage error was less than 12.0% for volume, 11.0% for heart area, 12.0% for CVR, and 12.0% for CTR.

**Table 3:** Percentage error of variability among the medical centers, %.

		Volume	Heart area	CVR	CTR
Second Xiangya Hospital	Intra-observer	7.7	7.3	6.1	6.9
	Inter-observer	11.3	10.7	11.9	11.3
Changsha Hospital for Maternal & Child Health Care	Intra-observer	7.9	6.8	7.7	7.5
	Inter-observer	10.1	10.2	10.9	11.8
Maternal & Child Health Hospital of Changde City	Intra-observer	6.3	8.3	7.2	8.1
	Inter-observer	11.5	10.9	11.1	10.7

### Comparison with prior studies

**Figure 4:**
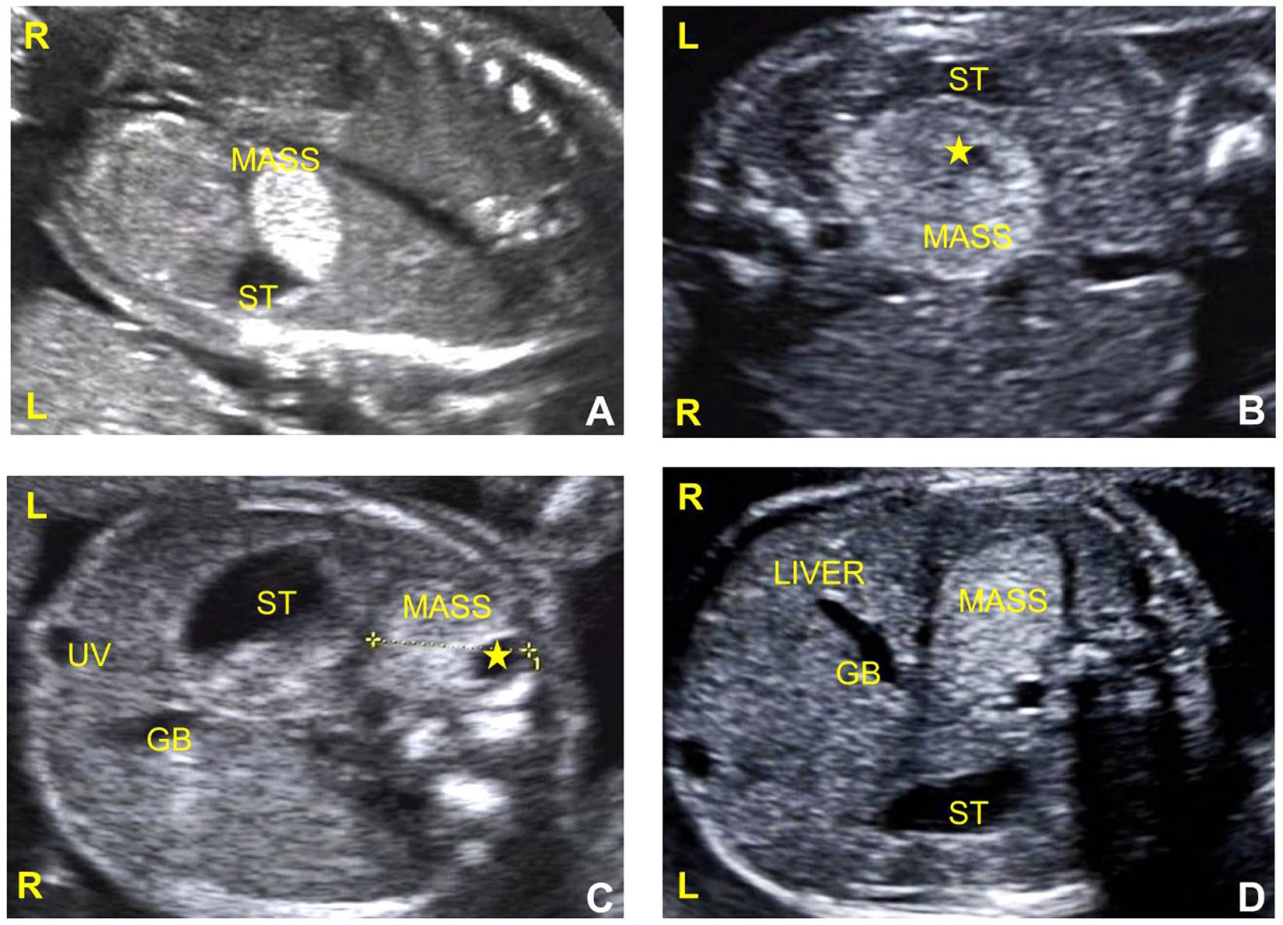
IEPS in gray-scale imaging of prenatal US. Ultrasound imaging of the upper fetal abdomen showing well-defined solid masses. (**a**) A homogenous and hyperechoic mass on the left side. (**b**,**c**) Heterogeneously hyperechoic solid masses with a small cystic component (asterisk) on the left side. (**d**) A homogenous and hyperechoic mass on the right side. ST, stomach; GB, gall bladder; UV, umbilical vein; L, left; R, right.

A comparison was conducted between the prenatal ultrasound characteristics, treatment, and outcomes of affected fetuses in the present study with that of prior studies (Table 4). The gestational ages at diagnosis of the present and prior studies were similar (24.2 ± 2.3 cf. 24.6 ± 6.1 weeks), as was the male-to-female ratio (3.3:1 cf. 4.3:1). In addition, there were no significant differences between the present study and prior ones regarding the ratio of lesions in the left suprarenal region and those in the right suprarenal region (Fig. 4). In terms of echotexture, the present study and prior reports were similar with regard to the ratio of homogenous and hyperechoic lesions, and heterogeneous masses with cysts.

**Table 4:** Ultrasound characteristics, treatment, & outcomes in present & prior studies, *n* (%).

			Present study	Prior studies	*P*
			2005–2017	1986–2017	
Subjects			21	43	—
US findings	Sidedness of the lesion: left		86% (18/21)	77% (33/43)	0.52
	Hyperechoic		67% (14/21)	47% (20/43)	0.18
	Hyperechoic with cysts		33% (7/21)	28% (12/43)	0.77
	Prenatal detection of feeding artery		86% (18/21)	7% (3/43)	0.00
		From ABD aorta	16	2	—
		From ABD aorta’s branches	2	1	—
	Associated anomalies		33% (7/21)	5% (2/43)	0.00
		Cardiovascular	5% (1/21)	0% (0/43)	0.33
		Diaphragmatic hernia	29% (6/21)	2% (1/43)	0.00
		Digestive tract	5% (1/21)	5% (2/43)	1.00
		Others	0% (0/21)	2% (1/43)	1.00
Treatment	Surgery		57% (12/21)	81% (35/43)	0.07
	Abortion		43% (9/21)	2% (1/43)	0.00
	Conservative observation		0% (0/21)	5% (2/43)	1.00
Outcomes	Survive		57% (12/21)	86% (37/43)	0.03
	Demise		43% (9/21)	5% (2/43)	0.00

ABD, abdominal; US, ultrasound.

It is notable that associated anomalies were detected in 33% (7/21) of fetuses with IEPS in the present study (Table 1), but only 5% (2/43, one with diaphragmatic hernia, gastric duplication cyst and bilateral choroid plexus cysts; and another with gastric duplication cyst) in prior studies (*P* < 0.01). In the present study, the prenatal rate of feeding arteries detected by color Doppler (86%) was significantly higher than that of prior studies (7%, *P* < 0.01). Two of the 43 patients (5%) in prior studies were followed late into childhood, with spontaneous regression of the lesion after birth in serial imaging studies. One patient in a prior study had several severe anomalies and died immediately after birth. In the present study, spontaneous regression was not found in any of the 21 patients.

## Discussion

Before 1986 there was no prenatal report of IEPS^9^, and it remains challenging to diagnose accurately due to the absence of clinical signs and symptoms in utero^10^. Our study investigated whether prenatal ultrasound imaging may improve the accuracy of IEPS diagnosis, and in addition identified the special characteristics of IEPS on ultrasonogram. This type of lesion was found in 0.01% of scanned patients from 2005 to 2017. We conclude that accurate prenatal diagnosis of IEPS relies on correct interpretation of sonographic features.

In gray-scale imaging, most IEPS lesions affected the left suprarenal region and involved homogenous hyperechoic solid masses with an ellipsoidal shape. Notably, the sliding sign was a unique feature of IEPS, defined as a mass that shifts during fetal breath movements or hiccups (both can be noticed as early as 10 weeks gestation^11,12^) and is non-synchronized relative to adjacent organs, such as the liver, stomach, spleen, kidney, and adrenal gland. To the best of our knowledge, there has been no previous report of the sliding sign associated with IEPS in the fetus. In the present study, most lesions had well-defined margins, and the sliding sign could be observed. Thus, it was easier to differentiate IEPS from other tumors or organs during fetal breath movements or hiccups.

Although the IEPS mass is supplied by the abdominal aorta, it is usually stable and rarely grows aggressively. Large masses in the fetal chest, such as an intrathoracic ELS, often lead to fetal hydrops and maldevelopment of the lungs^13^. In the present study, the heart area, CVR, and CTR were usually normal, and no ascites or hydrops were observed. This may be because IEPS had not progressed enough to cause high-output cardiac failure, or most lesions were located on the left side, far away from the inferior vena cava, which ensures a good venous return. Additionally, although some IEPS cases involved diaphragmatic hernia, the mass was under the diaphragm. This seems to block the diaphragmatic defect and prevent abdominal organs from further entering the thorax and compressing the fetal heart and lungs.

Another important essential ultrasound feature of IEPS in color Doppler imaging is the presence of feeding arteries arising from the abdominal aorta. Prenatal identification of the feeding artery by color Doppler was not reported until 1994^14^. Since then, feeding arteries have been detected more frequently, as the sensitivity of ultrasound has increased. Some studies reported that although systemic arterial flow is the most distinctive feature of IEPS on Doppler scan, it has high specificity but low sensitivity^8,15^. However, feeding arteries were identified in 86% of patients by using prenatal color Doppler in our study. The higher rate of detection of feeding arteries was due to the adoption of a more advanced ultrasound system, the higher sensitivity of Color Doppler, and thus greater ability to recognize the feeding arteries of IEPS. Our 3 medical centers have much experience in diagnosing IEPS. When IEPS is suspected, we focus on the relationship between the feeding artery and the abdominal aorta or its branches in multiple views, which are adjusted as necessary and not limited to the standard coronal, horizontal, and sagittal sections. Such observations allow more support for a correct diagnosis. Our study shows that feeding arteries can be identified prenatally by color Doppler, with convenience, safety, and low cost.

Diseases similar to IEPS include neuroblastoma, adrenal hematoma, and teratoma^6,8,16–18^, with neuroblastoma the most common. Neuroblastoma is not usually identified until the third trimester^17^, because sympathetic ganglion cells do not fully form until 18–20 weeks’ gestation^19^, whilst IEPS is usually detected in the second trimester^8^. In addition, IEPS is usually on the left side, neuroblastoma on the right^17^; IEPS appears as a homogenous and hyperechoic solid mass, while neuroblastoma is either a solid or a mixed-solid and cystic echotexture. Calcification is an essential feature of neuroblastoma^20^, but, other than Plattner *et al*.’s^13^ study, there have been no reports of calcification in IEPS. Plattner *et al*. suggested that calcification should not be the sole feature to rule out IEPS. Most IEPS appears with well-defined margins and the sliding sign, while neuroblastoma often invades the adjacent organs^8,21^. The feeding artery is an important differentiating feature to diagnose IEPS. Color Doppler imaging of neuroblastoma can reveal high vascularity in a solid/mixed mass or peripheral vascularization and no blood flow in a cystic mass^22–24^.

We recommend that any hyperechoic solid lesion detected in the upper abdomen (especially on the left) in the second trimester should prompt suspicion of IEPS. The sliding sign is important to differentiate IEPS from other tumors or organs. A clear feeding artery from the systemic circulation should confirm IEPS.

The natural course and consequences of IEPS is still not well known. Tuberculosis and nocardiosis have been reported in ELS cases^25^, but malignancy was rare (only 2 cases of squamous cell carcinoma had ever been reported^26,27^. ELS can also be complicated with other less common conditions such as hemothorax^28^ and torsion^29^. As a type of ELS, none of the IEPS cases in the present study showed any of these rare complications. This may be related to the sample size and the age of the fetuses or infants of the study population. Some previous studies have reported cases of spontaneous regression in IEPS. In the studies of Danielson and Sherman^30^ and Chowdhury *et al*.^31^, masses grew in utero and began to regress after birth, but the earliest to regress was 6 months after birth. However, in another case the mass continued to grow until surgical resection was performed at 2 years and 9 months of age^32^. In the present study, spontaneous regression was not noticed in any of the 21 cases. Long-term follow up may be required to observe the occurrence of spontaneous regression.

The treatment of IEPS is controversial. Since spontaneous regression is possible^31,33^, conservative therapy has been advocated. Today, surgical resection remains the mainstay treatment, because complete excision can eliminate the potential risks of many complications, such as infection and malignant degeneration^32^. The postsurgical (open and laparoscopic) outcomes of isolated IEPS in our study were satisfactory.

This study also has some limitations. Firstly, although our series is the largest of its kind to date, 21 fetuses is a small sample. To estimate more accurately the occurrence rate of fetal IEPS would require the participation of more medical centers. Secondly, the follow-up period was also brief. A larger series with long-term follow-up is needed to confirm the outcomes of surgical resection, and to determine the long-term post-birth consequences such as spontaneous regression.

## Methods

This study was approved by the institutional review board at The Second Xiangya Hospital, Changsha Hospital for Maternal & Child Health Care, and The Maternal and Child Health Hospital of Changde City. All methods were performed in accordance with the relevant guidelines and regulations. Written informed consent was obtained from the legal guardians.

### Ultrasound instruments at our centers

This study was performed in 3 prenatal diagnostic centers: The Second Xiangya Hospital; Changsha Hospital for Maternal & Child Health Care; and The Maternal and Child Health Hospital of Changde City. These hospitals are all local well-known hospitals with prenatal diagnostic centers in Hunan province, China. The fetal prenatal ultrasound examinations were performed using the following at each hospital: Siemens Acuson Sequoia 512 with 6C2 probe (2–6 MHz; Siemens Medical, Olympia, WA, USA), and GE Voluson 730 Expert and GE Voluson E8 Expert diagnostic system (GE Healthcare Ultrasound, Milwaukee, WI, USA) with RAB 4-8-D probe (4–8 MHz).

### Data collection from our database

We retrospectively searched the database of our 3 centers for cases of IEPS that had been prenatally diagnosed between March 2005 and December 2017. Only cases confirmed by autopsy or surgery pathology were included in the present study. For each case, baseline demographics, ultrasound findings, antenatal findings, fetal karyotypes, treatment, and outcomes were collected for further analysis. For ultrasound findings, the heart size, CVR, CTR, sonographic features of the lesion (size, shape, boundary, echotexture, and vascularity), and presence of hydrops were noted. To determine the heart size, heart area was measured and used to obtain Z-scores (to avoid the influence of gestational age) using previously published normative data^34^. Prior studies revealed that CVR was a useful measurement in diagnosing fetal congenital cystic adenomatoid malformation and PS, because CVR was found associated with fetal hydrops and postnatal symptoms^3,35^. Since IEPS is a type of PS, it is reasonable to speculate that CVR could also be adopted to assess the severity and prognosis of IEPS. The CVR was calculated by dividing the volume of IEPS by head circumference (HC), so that CVR = (Length × Width × Height × 0.52/HC^3,35^.

The diagnostic criteria for IEPS were the following: detected in the second trimester and continued to be present; homogenous and hyperechoic; with or without cystic components, and with no significant change in echotexture in follow-up serial exams; showed clear boundary and with the surrounding tissue with no obvious adhesion; a blood supply from the abdominal aorta or its branches; and a sliding sign (see Supplementary Video 1).

### Repeatability test for the present data

Two observers independently assessed the routine obstetrical and echocardiography parameters. To test intra-observer variability, a single observer analyzed the data twice on occasions separated by an interval of 1 month. To test inter-observer variability, a second observer analyzed the data without knowledge of the first observer’s measurements. Reproducibility was assessed as the mean percentage error (absolute difference divided by the mean of the 2 observations).

### Comparisons with previous studies

We also reviewed 43 prior cases that had been published between 1986 and 2017. For each case, baseline demographics, ultrasound findings, antenatal findings, treatment, and outcomes were collected and compared with the 21 cases from our database.

### Statistical analyses

Statistical analyses were performed using SPSS 17.0 software (SPSS, Chicago, IL). Data are presented as mean ± standard deviation for continuous variables and frequency (%) for categorical variables. Student’s *t*-test was used to compare continuous variables, and the chi-squared or Fisher’s exact test to compare categorical variables. A probability value of *P* < 0.05 was considered statistically significant.

## Conclusions

Prenatal ultrasound is widely used in the world and the diagnostic accuracy of IEPS has improved significantly. The principle signs that strongly indicate IEPS are the sliding sign and feeding vessels. The outcomes of surgical removal of the lesion were satisfactory in this study.

## Supplementary information

Supplementary Video 1

Supplementary information

## References

[1] Cooke CR (2006). Bronchopulmonary sequestration. Respir Care.

[2] Gezer S (2007). Pulmonary sequestration: a single-institutional series composed of 27 cases. J Thorac Cardiovasc Surg.

[3] Zhang H (2014). Retrospective study of prenatal diagnosed pulmonary sequestration. Pediatr Surg Int.

[4] Felker RE, Tonkin IL (1990). Imaging of pulmonary sequestration. AJR Am J Roentgenol.

[5] Savic B, Birtel FJ, Tholen W, Funke HD, Knoche R (1979). Lung sequestration: report of seven cases and review of 540 published cases. Thorax.

[6] Chan YF, Oldfield R, Vogel S, Ferguson S (2000). Pulmonary sequestration presenting as a prenatally detected suprarenal lesion in a neonate. J Pediatr Surg.

[7] Corbett HJ, Humphrey GM (2004). Pulmonary sequestration. Paediatr Respir Rev.

[8] Laje P, Martinez-Ferro M, Grisoni E, Dudgeon D (2006). Intraabdominal pulmonary sequestration. A case series and review of the literature. J Pediatr Surg.

[9] Mariona F, McAlpin G, Zador I, Philippart A, Jafri SZ (1986). Sonographic detection of fetal extrathoracic pulmonary sequestration. J Ultrasound Med.

[10] McCullagh M, MacConnachie I, Garvie D, Dykes E (1994). Accuracy of prenatal diagnosis of congenital cystic adenomatoid malformation. Arch Dis Child.

[11] de Vries JI, Visser GH, Prechtl HF (1986). Fetal behaviour in early pregnancy. Eur J Obstet Gynecol Reprod Biol.

[12] Popescu EA (2007). Magnetographic assessment of fetal hiccups and their effect on fetal heart rhythm. Physiol Meas.

[13] Plattner V (1995). Extra-lobar pulmonary sequestration with prenatal diagnosis. A report of 5 cases and review of the literature. Eur J Pediatr Surg.

[14] Maimon S, Siplovich L, Kaveh Z, Shalev E, Vigder F (1994). [Pulmonary sequestration detected by ultrasound]. Harefuah.

[15] Rosado-de-Christenson ML, Frazier AA, Stocker JT, Templeton PA (1993). From the archives of the AFIP. Extralobar sequestration: radiologic-pathologic correlation. Radiographics.

[16] Chen CP (1997). Clinical and perinatal sonographic features of congenital adrenal cystic neuroblastoma: a case report with review of the literature. Ultrasound Obstet Gynecol.

[17] Curtis MR (1997). Prenatal ultrasound characterization of the suprarenal mass: distinction between neuroblastoma and subdiaphragmatic extralobar pulmonary sequestration. J Ultrasound Med.

[18] White J, Chan YF, Neuberger S, Wilson T (1994). Prenatal sonographic detection of intra-abdominal extralobar pulmonary sequestration: report of three cases and literature review. Prenat Diagn.

[19] Lager DJ, Kuper KA, Haake GK (1991). Subdiaphragmatic extralobar pulmonary sequestration. Arch Pathol Lab Med.

[20] Brink DA, Balsara ZN (1991). Prenatal ultrasound detection of intra-abdominal pulmonary sequestration with postnatal MRI correlation. Pediatr Radiol.

[21] Alaish SM, Greenspon J, Strauch ED, Sun CC (2009). Intraabdominal pulmonary sequestration presenting with elevated urinary normetanephrine levels. J Pediatr Surg.

[22] Erol O, Suren D, Buyukkinaci Erol M (2013). Prenatal diagnosis of adrenal neuroblastoma: a case report with a brief review of the literature. Case Rep Obstet Gynecol.

[23] Houlihan C, Jampolsky M, Shilad A, Prinicipe D (2004). Prenatal diagnosis of neuroblastoma with sonography and magnetic resonance imaging. J Ultrasound Med.

[24] Menager N (2012). Prenatal diagnosis of atypical adrenal neuroblastoma with pulmonary metastases is possible: Impact on the assessment of prenatal prognosis. Diagn Interv Imaging.

[25] Kim HK (2005). Infected infradiaphragmatic retroperitoneal extralobar pulmonary sequestration: a case report. J Korean Med Sci.

[26] Bell-Thomson J, Missier P, Sommers SC (1979). Lung carcinoma arising in bronchopulmonary sequestration. Cancer.

[27] Hertzog P, Roujeau J, Marcou J (1963). Epidermoid cancer developed on a sequestration. J Fr Med Chir Thorac.

[28] Guska S (2004). Hemothorax caused by bleeding inside extralobar pulmonary sequestration in a patient on anticoagulation therapy. Med Arh.

[29] Huang EY, Monforte HL, Shaul DB (2004). Extralobar pulmonary sequestration presenting with torsion. Pediatr Surg Int.

[30] Danielson PD, Sherman NJ (2001). Laparoscopic removal of an abdominal extralobar pulmonary sequestration. J Pediatr Surg.

[31] Chowdhury M (2004). Spontaneous postnatal involution of intraabdominal pulmonary sequestration. J Pediatr Surg.

[32] Costa MR (2016). Atypical presentation of intra-abdominal extralobar pulmonary sequestration detected in prenatal care: a case report. Rev Paul Pediatr.

[33] Garcia-Pena P, Lucaya J, Hendry GM, McAndrew PT, Duran C (1998). Spontaneous involution of pulmonary sequestration in children: a report of two cases and review of the literature. Pediatr Radiol.

[34] Li X, Zhou Q, Huang H, Tian X, Peng Q (2015). Z-score reference ranges for normal fetal heart sizes throughout pregnancy derived from fetal echocardiography. Prenat Diagn.

[35] Crombleholme TM (2002). Cystic adenomatoid malformation volume ratio predicts outcome in prenatally diagnosed cystic adenomatoid malformation of the lung. J Pediatr Surg.

