# Supplementary material for: Prenatal diagnosis of fetal intraabdominal extralobar pulmonary sequestration: a 12-year 3-center experience in China
Source: Sci Rep. 2019 Jan 30;9:943. doi: 10.1038/s41598-018-37268-1 (PMC6353922; doi:10.1038/s41598-018-37268-1)
Supplement: Supplementary file 2 — Supplementary information [file 41598_2018_37268_MOESM2_ESM.docx]

**Prenatal diagnosis of fetal intraabdominal extralobar pulmonary sequestration: a 12-year 3-center experience in China**

Ganqiong Xu^1^, Jiawei Zhou^1^, Shi Zeng^1^, Ming Zhang^1^, Zhu Ouyang^1^, Yili Zhao^1^, Hongxia Yuan^2^, Lili Tong^3^, Chan Yin^3^, Qichang Zhou^1*^

^1^Department of Ultrasound Diagnosis, The Second Xiangya Hospital, Central South University, Changsha, Hunan 410011, China

^2^Department of Ultrasound, Changsha Hospital for Maternal & Child Health Care

^3^Department of Ultrasound, The Maternal and Child Health Hospital of Changde City

^*^Correspondence and requests for materials should be addressed to Q.C.Z., Department of Ultrasound, The Second Xiangya Hospital, Central South University, No. 139 Middle Renmin Road, Changsha, Hunan 410011, China

E-mail: zhouqc@csu.edu.cn

Phone number: 86-0731-85292140. Fax: 86-0731-85292140

**Supplementary Video 1. Sliding sign.** A mass is shifting during fetal hiccups non- synchronized with adjacent organs
